# Experience and expectations of patients on weight loss: The Learning Health System Network Experience

**DOI:** 10.1002/osp4.364

**Published:** 2019-08-22

**Authors:** R. S. DeJesus, K. W. Bauer, D. P. Bradley, I. Haller, S. M. Bradley, D. R. Schroeder, J. St. Sauver, S. M. Phelan, I. T. Croghan

**Affiliations:** ^1^ Department of Medicine Mayo Clinic Rochester MN USA; ^2^ Department of Nutritional Sciences University of Michigan School of Public Health Ann Arbor MI USA; ^3^ Diabetes and Metabolism Research Center, Division of Endocrinology, Diabetes & Metabolism, Department of Internal Medicine The Ohio State University Columbus OH USA; ^4^ Essentia Institute of Rural Health, Essential Health Duluth MN USA; ^5^ Center for Healthcare Delivery Innovation Minneapolis Heart Institute and Minneapolis Heart Institute Foundation Minneapolis MN USA; ^6^ Department of Health Sciences Research Mayo Clinic Rochester MN USA; ^7^ Robert D. and Patricia E. Kern Center for the Science of Health Care Delivery Mayo Clinic Rochester MN USA

**Keywords:** Patient expectations, primary care, weight loss

## Abstract

**Objective:**

Weight perception and degree of confidence in achieving healthy lifestyle can be determinants of engagement in obesity interventions. This study explored patients' perceived need for weight loss and the degree of self‐confidence in ability to lose weight and sought to identify factors associated with patients' self‐confidence in ability to lose weight.

**Methods:**

The authors analysed data from a survey mailed to primary care patients within five sites of the Learning Health Systems Network that explored participants' prior experience with weight management.

**Results:**

Among the 2,263 participants who completed the survey section on ‘Patients’ Experience with Weight Management’, perceived need to lose 51 lb or more was statistically significant among those with class III obesity compared with other body mass index (BMI) groups (*p* value < 0.001). Reported desire to lose weight was also significantly higher among those with the highest BMI than those who were overweight (*p* value < 0.001). However, this same group had the lowest belief in ability to lose weight (*p* value < 0.001). In a multiple regression analysis, female gender, higher BMI and need to lose >10 lb were each independently associated with less belief in being able to lose weight.

**Conclusions:**

Patients had varying perceptions on weight loss; those with category III obesity had the highest desire to lose weight but had the least confidence in ability to lose weight. Higher BMI, female gender and need to lose >10 lb were associated with decreased self‐confidence in ability to lose weight.

## 1 Introduction

In 2016, more than 650 million adults worldwide aged 18 years and older have a body mass index (BMI) in the obese category. The worldwide prevalence of obesity nearly tripled between 1975 and 2016; it has been linked to more deaths than has underweight.^1^ The US Preventive Services Task Force, the US National Institutes of Health, the American Heart Association, the American College of Cardiology and the Obesity Society have independently developed guidelines on assessment and management of obesity that primarily involve multicomponent behavioural or lifestyle interventions.^2^, ^3^, ^4^

Weight perceptions and confidence in one's ability to achieve a healthy lifestyle can be determinants of engagement in overweight and obesity interventions. A prior study performed in a large institution showed that while individuals with higher BMI were more likely to report that their physician had told them that they had overweight, such conversations were less likely to change their personal view of their weight or motivate them to lose weight.^5^ A deeper insight on patient experience and expectations regarding weight loss can therefore assist clinicians in planning patient‐centred intervention strategies and refining existing interventions to better ensure outcome success.

Studies evaluating perceptions of individuals who have overweight or obesity had consisted of small size and had yielded contrasting observations, from those who had realistic perception of obesity and recognized the need to lose weight, to those whose perception of obesity was below its true prevalence.^6^, ^7^ To address this limitation and obtain a more robust insight on the personal experiences and expectations regarding weight loss among patients who have overweight and obesity, data were analysed from a survey of primary care patients within the Learning Health Systems Network (LHSNet). The LHSNet survey was conducted to identify barriers to successful weight loss and patients' expectations regarding primary care physicians' assistance in weight management.^8^ A section of this survey specifically explored participants' prior experience with weight management, their perceived need for weight loss and the degree of self‐readiness and self‐confidence in ability to lose weight, which is the primary focus of our study. The investigators further sought to identify factors that were associated with patients' self‐confidence in ability to lose weight.

## 2 Methods

The LHSNet is composed of nine sites and includes data on approximately 10 million patients. Details of the LHSNet structure and sites have been described elsewhere.^9^, ^10^ Each LHSNet site hosts a local common data model (CDM) DataMart and share common data elements across sites. The LHSNet CDM contains outcome data on patient cohorts including the ‘weight cohort’. The building of the weight cohort centred on cohorts of children and adults across the LHSNet that were assembled and identified as having overweight or obesity based on PCORnet obesity algorithm, focusing on individuals with at least one other height and weight measurement in the previous 5 years. Weights of pregnant women were excluded from 6 months before to 3 months after delivery date. Patients within the weight cohort at five of the nine LHSNet sites served as the sampling population for the survey. These five sites contributed the majority of patients within the weight cohort. The study was deemed exempt by Mayo Clinic Institutional Review Board, which served as the institutional review board of record for the participating study sites. Protocol‐approved passive consent was obtained from all study participants before study initiation. Five LHSNET sites participated in this study.

### 2.1 Survey instrument

The Weight Management Survey was developed by the LHSNet weight cohort work group on the basis of previously developed questions/scales of patient beliefs, behaviours and experiences.^11^, ^12^, ^13^, ^14^, ^15^, ^16^ The development and detailed features of the survey had been described in a recently published study.^8^ The survey had five main sections, one of which was Patients' Experience with Weight Management. This section consisted of seven items with combined closed‐ and open‐ended questions. The first item asked the question ‘Have you gained or lost weight recently’; for a ‘yes’ response to weight loss, an additional open‐ended question asked how weight loss was achieved. Items 2 and 3 asked ‘Do you think you need to lose weight (yes/no)’ and ‘How much do you think you need to lose (10 lbs. or less, 11‐25 lbs., 26‐50 lbs., 50 lbs. or more)’, respectively. Items 4 and 5 asked the respondents to rate, on a Likert scale of 1–10, how much they desire to lose weight and how much they believe they can lose weight, respectively. For items 6 and 7, respondents checked off listed factors that ‘would motivate you to seek weight information’ and ‘major barriers to your losing weight’. A copy of the survey is available upon request from the authors.

The survey was purposefully developed to be brief and anonymous. Surveys were delivered and returned via US Postal Service. Only one mailing was performed.

### 2.2 Data collection

Each of the five participating LHSNet sites ran a query on its local CDM DataMart to identify patients who fit study criteria. Eligible patients were 18 years or older with available weight and height (for BMI calculation). The query randomly selected 4,000 patients per site who met the criteria with additional stratification of 1,000 patients per BMI category: overweight (BMI, 25–29.9 kg m^−2^), obesity class I (BMI, 30–34.9 kg m^−2^), obesity class II (BMI, 35–39.9 kg m^−2^), obesity class III (BMI, >40 kg m^−2^), totalling 20,000 for the network.

From the 20,000 selected patients, 19,964 had valid addresses. The surveys were mailed during a 4‐week period from 27 October 2017, through 22 November 2017; data collection lasted 1 months from 27 October 2017 through 1 March 2018. Three hundred thirteen mailed surveys were returned by the US Postal Service as ‘undeliverable’. Of the remaining 19,652 delivered surveys, 2,799 (14.2%) were returned as completed with an additional 1,033 (5.2%) refusal to participate. The Mayo Clinic Survey Research Center received and entered the data from all returned surveys using a dual‐data‐entry system. A detailed flow diagram of study participants has been presented elsewhere.^8^

### 2.3 Data analysis

Quantitative data on experience and expectations of patients were analysed using chi‐square tests to compare categorical variables across BMI category groups and analysis of variance to compare continuous variables across groups. A single multiple linear regression model using the respondents' self‐reported belief in their ability to lose weight as dependent variable was used to analyse data for potentially associated characteristics.

Qualitative data from patients' responses on weight loss measures that had been used in the past were grouped into major themes.

## 3 Results

Among the 2,263 participants who completed the survey section on ‘Patients’ Experience with Weight Management’, 648 (29%) had overweight, 619 (27%) had class I obesity, 474 (21%) had class II obesity and 522 (23%) had class III obesity. Demographic characteristics differed by weight category; persons in the class III obesity category were younger, were more likely to be female, were more likely to be non‐Caucasian, and have some college education or more (Table 1).

**Table 1:** Respondents' characteristics

Characteristic	Body mass index, kg m^−2^				p ^a^
	25.0–29.9 (N = 648^b^)	30.0–34.9 (N = 619^b^)	35.0–39.9 (N = 474^b^)	≥40.0 (N = 522^b^)	
Age, years	62.0 ± 14.7	60.5 ± 13.6	58.4 ± 13.2	54.1 ± 14.2	<0.001
Sex					<0.001
Male	309 (47.7)	280 (45.2)	166 (35.0)	134 (25.7)	
Female	339 (52.3)	339 (54.8)	308 (65.0)	388 (74.3)	
Race					0.016
White, non‐Hispanic	591 (93.5)	554 (92.9)	420 (91.3)	453 (88.6)	
Other	41 (6.5)	42 (7.1)	40 (8.7)	58 (11.4)	
Education					<0.001
High school graduate or less	123 (19.2)	118 (19.4)	95 (20.5)	113 (22.2)	
Some college	204 (31.9)	210 (34.5)	170 (36.6)	229 (44.9)	
Four year college degree or more	313 (48.9)	280 (46.1)	199 (42.9)	168 (32.9)	

Age was compared across groups using analysis of variance, and categorical variables were compared across groups using the chi‐square test.

Due to missing data, the sum of the response categories for a given characteristic may not equal the total number of respondents.

When asked if they have lost weight recently, 920 participants positively responded to the question. Major themes identified as measures taken to lose weight were dietary interventions (diet modification and diet programmes), exercise, change in eating/physical activity patterns and surgery, with the largest percentage of responders having attempted dietary management (53.15%, *N* = 489). Weight loss programmes, medications and surgery were utilized more by those in the higher BMI categories; 23 of 37 participants who had bariatric surgery and 15 of 31 participants who took weight loss medications had a BMI of 35 and above (Table 2).

**Table 2:** Weight loss method themes by BMI

Weight loss method	BMI 25–29.9 (N = 257)	BMI 20–34.9 (N = 256)	BMI 35–39.9 (N = 185)	BMI 40 and up (N = 222)
Diet/diet programme ‘ate less/ate different’, ‘change eating habit’, ‘count calories/portion control’	154 (59.9%)	138 (53.9%)	90 (48.6%)	107 (48.1%)
Exercise ‘walk/jog’, ‘sports/swim/train’	80 (31.1%)	80 (31.2%)	43 (23.2%)	45 (20.2%)
Weight loss medication	10 (3.9%)	6 (2.3%)	5 (2.7%)	10 (4.5%)
Weight loss surgery ‘bariatric surgery’	4 (1.5%)	10 (3.9%)	9 (4.8%)	14 (6.3%)
Others (illness, weight loss programme, stress) ‘got sick, had cancer’, ‘depressed; stressed’	77 (29.9%)	77 (30.0%)	74 (40.0%)	81 (36.4%)

Percentage may not add up to 100% because categories are not mutually exclusive.

BMI, body mass index.

Table 3 describes the respondents' weight management expectations by BMI category groups. Higher proportion of those with class III obesity had expectations regarding the need to lose weight and the need to lose >50 lb than the other BMI groups (*p* value < 0.001). Lower BMI categories were associated with perceived need for lesser weight loss.

**Table 3:** Weight management expectations

Characteristic	Body mass index, kg m^−2^				p ^a^
	25.0–29.9 (N = 648^b^)	30.0–34.9 (N = 619^b^)	35.0–39.9 (N = 474^b^)	≥ 40.0 (N = 522^b^)	
Do you think you need to lose weight?					<0.001
No	162 (25.0)	36 (5.8)	7 (1.5)	4 (0.8)	
Yes	486 (75.0)	583 (94.2)	467 (98.5)	518 (99.2)	
How much do you think you need to lose?^b^					<0.001
10 lb or less	155 (32.0)	41 (7.1)	1 (0.2)	3 (0.6)	
11 to 25 lb	267 (55.0)	248 (42.9)	61 (13.1)	12 (2.3)	
26 to 50 lb	62 (12.8)	240 (41.5)	197 (42.4)	53 (10.2)	
51 lb or more	1 (0.2)	49 (8.5)	206 (44.3)	449 (86.9)	
How much do you desire to lose weight? (0–10)^b^					<0.001
Mean ± SD	7.7 ± 2.2	7.9 ± 2.0	8.4 ± 2.0	8.7 ± 1.8	
Median (25th, 75th)	8 (6, 10)	8 (7, 10)	9 (7, 10)	10 (8, 10)	
How much do you believe you can lose weight? (0–10)^b^					<0.001
Mean ± SD	7.7 ± 2.2	7.1 ± 2.4	6.6 ± 2.5	6.5 ± 2.7	
Median (25th, 75th)	8 (6, 10)	7 (5, 9)	7 (5, 9)	7 (5, 9)	
What are the major barriers to your losing weight?^b^					
Not enough time to do physical activity	113 (23.3)	111 (19.0)	92 (19.7)	94 (18.2)	0.196
Medical condition that limits ability to do physical activity	106 (21.8)	180 (30.9)	162 (34.7)	250 (48.3)	<0.001
Do not have access to healthy food choices	12 (2.5)	22 (3.7)	20 (4.3)	46 (8.9)	<0.001
Do not have access to a facility to do physical activity	32 (6.6)	54 (9.3)	42 (9.0)	79 (15.3)	<0.001
Cannot afford a gym membership	51 (10.5)	107 (18.4)	109 (23.3)	171 (33.0)	<0.001
Pressure from my family or peers to not lose weight	6 (1.2)	8 (1.4)	7 (1.5)	16 (3.1)	0.086
Food cravings that prevent me from losing weight	192 (39.5)	234 (40.1)	230 (49.3)	262 (50.6)	<0.001
Not sure which foods to eat to help lose weight	47 (9.7)	101 (17.3)	67 (14.4)	91 (17.6)	0.001
Not enough time to do physical activity	113 (23.3)	111 (19.0)	92 (19.7)	94 (18.2)	0.196
Medical condition that limits ability to do physical activity	106 (21.8)	180 (30.9)	162 (34.7)	250 (48.3)	<0.001
Do not have access to healthy food choices	12 (2.5)	22 (3.7)	20 (4.3)	46 (8.9)	<0.001
Do not have access to a facility to do physical activity	32 (6.6)	54 (9.3)	42 (9.0)	79 (15.3)	<0.001

Categorical variables were compared across groups using the chi‐square test, and continuous variables were compared across groups using analysis of variance.

Data are summarized only for patients who indicated that they think they need to lose weight.

To the survey questions that asked the responders to rate, on a scale of 1–10, their desire to lose weight and belief on their ability to lose weight, those with BMI of 40 and higher reported significantly higher desire to lose weight than those with BMI in overweight category (8.7 ± 1.8 SD vs. 7.7 ± 2.2 SD, *p* value < 0.001). However, the same group that had the greatest desire to lose weight had the lowest belief in ability to lose weight (6.5 ± 2.7 SD vs. 7.7 ± 2.2 SD, *p* value < 0.001).

In a multiple regression analysis, being female, having higher BMI and needing to lose >10 lb were independently associated with lesser belief in being able to lose weight. Similarly, the presence of medical condition that limits physical activity, food cravings and uncertainty on food choices was inversely associated with belief in ability to lose weight. Greater desire for weight loss was positively associated with increased belief in ability to lose weight (Table 4).

**Table 4:** Characteristics potentially associated with belief in being able to lose weight

Characteristic	Estimate	(95% CI)	p
Age, per 10 years	−0.0	(−0.1, 0.1)	0.553
Female	−0.7	(−1.0, −0.5)	<0.001
White, non‐Hispanic	−0.5	(−0.9, −0.1)	0.013
Desire to lose weight (0–10), per 1 point	0.4	(0.4, 0.5)	<0.001
Education			0.601
High school graduate or less	Reference		
Some college	0.0	(−0.3, 0.3)	
Four year college degree or more	−0.1	(−0.4, 0.2)	
Body mass index, kg m^−2^			<0.001
25.0–29.9	Reference		
30.0–34.9	−0.5	(−0.8, −0.2)	
35.0–39.9	−0.9	(−1.3, −0.5)	
≥ 40.0	−0.9	(−1.4, −0.5)	
How much do you think you need to lose?			0.036
10 lb or less	Reference		
11 to 25 lb	−0.3	(−0.7, 0.1)	
26 to 50 lb	−0.4	(−0.9, 0.0)	
51 lb or more	−0.8	(−1.3, −0.2)	
Barriers to losing weight			
Not enough time to do physical activity	−0.1	(−0.4, 0.2)	0.388
Medical condition that limits ability to do physical activity	−0.3	(−0.5, −0.0)	0.022
Do not have access to healthy food choices	0.1	(−0.4, 0.7)	0.579
Do not have access to a facility to do physical activity	0.0	(−0.4, 0.4)	0.929
Cannot afford a gym membership	−0.2	(−0.5, 0.1)	0.175
Pressure from my family or peers to not lose weight	−0.2	(−1.0, 0.5)	0.590
Food cravings that prevent me from losing weight	−0.5	(−0.7, −0.3)	<0.001
Not sure which foods to eat to help lose weight	−0.4	(−0.7, −0.1)	0.009

Data were analysed using a single multiple linear regression model with the respondent's self‐reported belief in their ability to lose weight (0–10) as the dependent variable. For each explanatory variable, the estimated regression coefficient is presented along with a corresponding 95% confidence interval. For categorical variables with >2 categories, the *p* value corresponds to the multiple degree of freedom test comparing across all categories simultaneously.

## 4 Discussion

Results from this study yielded several observations that were associated with participants' weight loss experiences and expectations. The majority of respondents who were able to achieve weight loss did so through lifestyle measures, mainly diet modification. Indeed, data adequately support the effectiveness of behaviour‐based interventions with or without pharmacologic agents for weight loss.^17^, ^18^ A statistical comparison of the weight loss methods by BMI category was not performed; however, a trend towards non‐behavioural methods among participants in higher BMI categories was observed.

Patients who had overweight or had obesity recognized the need for weight loss. Those with higher BMI perceived the need to lose more weight and expressed greater desire to lose weight. More than 50% of those with class II obesity desired to lose at least 26 lb, and majority of those with class III obesity would like to lose over 50 lb. The average behavioural weight loss programmes that focused on lifestyle measures (nutritional counselling, physical activity and improvement in sleep pattern) result in 5–7% weight loss; a slightly higher percentage of weight loss (8–10%) had been achieved in more intensive, comprehensive programmes.^18^, ^19^, ^20^ Hence, a weight loss goal of ≥50 lb may not be generally achievable through non‐surgical and non‐pharmacological interventions. This unrealistic weight loss goals and expectations had previously been seen among patients who had sought behavioural, pharmacological and surgical treatment for obesity^21^, ^22^, ^23^, ^24^ and had been reported as a barrier to effective weight loss intervention outcomes.^25^ Assistance with goal setting, problem solving and a multi‐faceted weight loss intervention programme with regular feedbacks and motivational support would be helpful in moving patients forward to successfully achieving realistic weight loss goals.

Interestingly, the same group of patients who would benefit most from weight loss interventions, i.e. those with higher BMI and those who felt the need to lose >10 lb, had the least confidence in their ability to lose weight. While an independent correlation between greater desire to lose weight and increased belief in ability to lose weight was seen in a multiple regression analysis, the perceived confidence in being able to lose weight decreased with higher BMI. One plausible explanation would be the increased likelihood that this group of patients may have already tried several weight loss measures but were unsuccessful in their attempts at losing weight, which consequently decreased their perceived confidence in their ability to lose weight. This had been observed even among well‐motivated individuals who have had several unsuccessful quit attempts at smoking cessation.^26^ Frustration with multiple failed efforts at weight loss eventually prompted most patients to seek bariatric surgery.^27^ The survey did not ask responders for the frequency of attempts at weight loss; this is a gap that would be interesting to investigate further.

This inverse relationship between desire and confidence to lose weight may also partly be due to ambivalence to come forward and seek help due to stigma‐related cognitions commonly experienced by those with higher BMI. In a semi‐structured interview conducted among a small group of individuals with diverse backgrounds and levels of obesity, reluctance in presenting their concerns to their primary care physicians stemmed from self‐consciousness to their features and uncertainty as to how they would be perceived.^28^ This highlights one the many challenges faced by individuals with obesity. While a discussion on stigmatization associated with overweight and obesity is beyond the scope of this paper, it is well reported in literature to impact motivation for lifestyle behaviour change, self‐confidence and obesity management outcomes.^29^, ^30^, ^31^

In his social cognitive theory, Albert Bandura defined self‐efficacy as confidence in one's ability to perform a task that affects whether thought is translated into action.^32^ Individuals are likely to pursue a course of action if perceived to be attainable within their capabilities. Judgments of self‐efficacy also determine how much effort people will expend and how long they will persist in the face of obstacles or aversive experiences. The role of self‐efficacy in driving behavioural changes and predicting success in weight loss interventions has been increasingly recognized in recent years.^33^, ^34^ Coaching and other strategies that improve self‐efficacy have been shown to positively impact lifestyle behaviours that potentially contribute to sustained weight loss and challenge us to rethink our approach to weight loss management.^35^, ^36^, ^37^

An association between being female and decreased confidence in ability to lose weight was also seen in a multiple regression analysis. Several factors may have contributed to this observation. Women not only tend to experience transitional events in life that trigger weight gain such as pregnancy and menopause but also are more vulnerable than men to weight stigmatization.^38^ There is also a bidirectional relation between depression and obesity that has been observed more among women than men that may impact one's self‐confidence.^39^, ^40^ Likewise, women more likely have had prior weight loss attempts that might have been unsuccessful, thereby lowering their self‐confidence in ability to lose weight. Further studies that explore gender associated aspects of obesity may be needed.

The major strength of this study is the inclusion of five diverse sites and large sample groups. The surveys were also anonymously completed, which helped reduce potential barriers in patients' participation at reporting personal perceptions and experiences. This anonymity, however, prevented linking the participants' data to health records, which is one limitation of this study. The survey asked the participants if they had previously lost weight and what measures helped them to lose weight. It did not ask how much weight loss was achieved or any behavioural change made as well as the number of attempts at weight loss and measures used for weight loss maintenance, which would have provided additional relevant information regarding patients' experience. The 12% response rate may have resulted in a study sample not being representative of the general population of patients with overweight and obesity receiving health care at study sites; however, it should be noted that response rates of mailed surveys have steadily declined in recent years.^41^ Participants were also mainly non‐Hispanic Whites; hence, results may not be generalized to other ethnic groups.

## 5 Summary

Participants in this study had varying expectations regarding weight loss, both on perceived need and on belief in ability to lose weight. Those with higher BMI had perceived need for greater, potentially unrealistic weight loss but also reported being least confident at their ability to lose weight. In a multiple regression analysis, being female, having higher BMI and needing to lose 11 lb or more were independently associated with lesser belief in being able to lose weight. Results from this study affirmed the importance of incorporating goal‐setting, problem‐solving and self‐efficacy skills through motivational interviewing, coaching or other strategies in weight management programmes.

## Conflict of Interest Statement

The authors report no competing interests.

## FUNDING

This research was supported in part by the Patient‐Centered Outcomes Research Institute (PCORI) CDRN‐1501‐26638 (V. Roger, PI) and by the Mayo Clinic Robert D. and Patricia E. Kern Center for the Science of Health Care Delivery.

## References

[1] World Health Organization . Overweight and Obesity. http://www.who.int/news‐room/fact‐sheets/detail/obesity‐and‐overweight. Accessed December 8, 2018.

[2] Moyer VA , U.S. Preventive Services Task Force . Screening for and management of obesity in adults: U.S. Preventive Services Task Force recommendation statement. Ann Intern Med 2012 Sep; 157: 373–378. 22733087 10.7326/0003-4819-157-5-201209040-00475

[3] Jensen MD , Ryan DH , Donato KA , et al. Guidelines (2013) for managing overweight and obesity in adults. Obesity 2014; 22: S1–S410. 10.1002/oby.20819 24961822

[4] US Preventive Services Task Force , Curry SJ , Krist AH , Owens DK , et al. Behavioral weight loss interventions to prevent obesity‐related morbidity and mortality in adults: US Preventive Services Task Force Recommendation Statement. JAMA 2018 Sep; 320: 1163–1171. 30326502 10.1001/jama.2018.13022

[5] Croghan IT , Huber JM , Hurt RT , et al. Patient perception matters in weight management. Prim Health Care Res Dev 2018 Mar; 19: 197–204. 29157321 10.1017/S1463423617000585 PMC6452950

[6] Mawardi G , Kirkland EB , Zhang J , et al. Patient perception of obesity versus physician documentation of obesity: a quality improvement study. Clin Obes 2019 Feb; 9: e12303. 30816010 10.1111/cob.12303

[7] Nelson DA , Rufflo LA , Dyer AJ , Nelson KH . Patient perceptions of weight loss: implications for patients, providers and trainees. Int J Pyschiatry Med 2016 May; 51: 325–336. 10.1177/0091217416659269 27497453

[8] Croghan IT , Phelan SM , Bradley DP , et al. Needs assessment for weight management: The LEARNING Health System Network Experience. Mayo Clin Proc Inn Qual Out 2018 Sept; 2: 324–335. 10.1016/j.mayocpiqo.2018.08.001 PMC6260476 30560234

[9] Finney Rutten LJ , Alexander A , Embj PJ , et al. Patient‐Centered Network of Learning Health Systems: developing a resource for clinical translational research. J Clin Transl Sci 2017; 1: 40–44. 28515960 10.1017/cts.2016.11 PMC5413962

[10] LHSNet. LHSNet . Patient Centered Network of Learning Health Systems . https://www.pcori.org/research‐results/2015/patient‐centered‐network‐learning‐health‐systems‐lhsnet Accessed December 8, 2018.

[11] Potter MB , Vu JD , Croughan‐Minihane M . Weight management: what patients want from their primary care physicians. J Fam Pract 2001; 50: 513–518. 11401737

[12] National Cancer Institute . HINTS: Health Information Trends Survey.

[13] Drury CA , Louis M . Exploring the association between body weight, stigma of obesity, and health care avoidance. J Am Acad Nurse Pract 2002; 14: 554–561. 12567923 10.1111/j.1745-7599.2002.tb00089.x

[14] Myers A , Rosen JC . Obesity stigmatization and coping: relation to mental health symptoms, body image, and self‐esteem. Int J Obes Relat Metab Disord 1999; 23: 221–230. 10193866 10.1038/sj.ijo.0800765

[15] Kroenke K , Spitzer RL , Williams JB . The Patient Health Questionnaire‐2: validity of a two‐item depression screener. Med Care 2003; 41: 1284–1292. 14583691 10.1097/01.MLR.0000093487.78664.3C

[16] Saltzer EB . The weight locus of control (WLOC) scale: a specific measure for obesity research. J Pers Assess 1982; 46: 620–628. 7161695 10.1207/s15327752jpa4606_11

[17] LeBlanc EL , Patnode CD , Webber EM , Redmond N , Rushkin M , O'Connor EA . Behavioral and pharmacotherapy weight loss interventions to prevent obesity‐related morbidity and mortality in adults: an updated systematic review for the U.S. Preventive Services Task Force. Agency for Healthcare Research and Quality. 2018 Sep. Report No: 18–05239‐EF‐1. 30354042

[18] Johnston BC , Kanters S , Bandayrel K , et al. Comparison of weight loss among named diet programs in overweight and obese adults: a meta‐analysis. JAMA 2014 Sep; 312: 923–933. 25182101 10.1001/jama.2014.10397

[19] Marek RJ , Coulon SM , Brown JD , et al. Characteristics of weight loss trajectories in a comprehensive lifestyle intervention. Obesity (Silver Spring) 2017 Dec: 2067–2067. 10.1002/oby.21942 29086487

[20] Lv N , Azar KMJ , Rosas LG , Wulfovich S , Xiao L , Ma J . Behavioral lifestyle interventions for moderate and severe obesity: a systematic review. Prev Med 2017 Jul; 100: 180–193. 28450123 10.1016/j.ypmed.2017.04.022 PMC5503454

[21] Foster GD , Wadded TA , Vogt RA , Brewer G . What is a reasonable weight loss? Patients' expectations and evaluations of obesity treatment outcomes. J Consult Clin Psychol 1997 Feb; 65: 79–85. 9103737 10.1037//0022-006x.65.1.79

[22] Fabricatore AN , Wadden TA , Womble LG , et al. The role of patients' expectations and goals in the behavioral and pharmacological treatment of obesity. Int J Obes (Lond) 2007 Nov; 31: 1739–1745. 17471295 10.1038/sj.ijo.0803649

[23] Wee CC , Hamel MB , Apovian CM , et al. Expectations for weight loss and willingness to accept risk among patients seeking weight loss surgery. JAMA Surg 2013; 148: 264–271. 23553327 10.1001/jamasurg.2013.1048 PMC4510944

[24] Kaly P , Orellana S , Torrella T , Takagishi C , Saff‐Koche L , Murr MM . Unrealistic weight loss expectations in candidates for bariatric surgery. Surg Obes Relat Dis 2008 Jan‐Feb; 4: 6–10. 18201668 10.1016/j.soard.2007.10.012

[25] Burgess E , Hassmen P , Pumpa KL . Determinants of adherence to lifestyle intervention in adults with obesity: a systematic review. Clin Obes 2017 Aug; 7: 123–135. 28296261 10.1111/cob.12183

[26] Smit ES , Hoving C , Schelleman‐Offermans K , West R , de Vries H . Predictors of successful and unsuccessful quit attempts among smokers motivated to quit. Addict Behav 2014 Sep; 39: 1318–1324. 24837754 10.1016/j.addbeh.2014.04.017

[27] Gibbons LM , Sarwer DB , Crerand CE , et al. Previous weight loss experiences of bariatric surgery candidates: how much have patients dieted prior to surgery? Obesity 2006 Mar; 14: 70S–76S. 16648597 10.1038/oby.2006.285

[28] Brown I , Thompson J , Tod A , Jones G . Primary care support for tackling obesity: a qualitative study of the perceptions of obese patients. Br J Gen Pract 2006; 56: 666–672. 16953998 PMC1876632

[29] Vartanian LR , Porte AM . Weight stigma and eating behavior: a review of the literature. Appetite 2016 Jul; 102: 3–14. 26829371 10.1016/j.appet.2016.01.034

[30] Phelan SM , Burgess DJ , Yeazel MW , Hellerstedt WL , Griffin JM , van Ryn M . Impact of weight bias and stigma on quality of care and outcomes for patients with obesity. Obes Rev 2015 Apr; 16: 319–326. 25752756 10.1111/obr.12266 PMC4381543

[31] Tomiyama AJ , Carr D , Granberg EM , et al. How and why weight stigma drives the obesity ‘epidemic’ and harms health. BMC Med 2018 Aug; 16: 123. 30107800 10.1186/s12916-018-1116-5 PMC6092785

[32] Bandura A . Self‐efficacy mechanism in human agency. Am Psychol 1982 Feb; 37: 122–147.

[33] Teixeira PJ , Carraça EV , Marques MM , et al. Successful behavior change in obesity interventions in adults: a systematic review of self‐regulation mediators. BMC Med 2015 Apr; 13: 84. 25907778 10.1186/s12916-015-0323-6 PMC4408562

[34] Flølo TN , Tell GS , Kolotkin RL , et al. Eating self‐efficacy as predictor of long‐term weight loss and obesity‐specific quality of life after sleeve gastrectomy: a prospective cohort study. Surg Obes Relat Dis 2019 Feb; 15: 161‐167. 30709748 10.1016/j.soard.2018.12.011

[35] DeJesus RS , Clark MM , Finney‐Rutten LJ , et al. Impact of a 12‐week wellness coaching on self‐care behaviors among primary care adult patients with prediabetes. Prev Med Rep 2018 Feb; 10: 100–105. 29850394 10.1016/j.pmedr.2018.02.012 PMC5966585

[36] Painter SL , Ahmed R , Kushner RF , et al. Expert coaching in weight loss: retrospective analysis. J Med Internet Res 2018 Mar; 20: e92. 29535082 10.2196/jmir.9738 PMC5871741

[37] Dayan PH , Sforzo G , Boisseau N , Pereira‐Lancha LO , Lancha AH Jr . A new clinical perspective: treating obesity with nutritional coaching versus energy‐restricted diets. Nutrition 2019 Apr; 60: 147–151. 30586658 10.1016/j.nut.2018.09.027

[38] Audet M , Baillot A , Vibarel‐Rebot N . Female obesity and physical activity: a better understanding of the stakes linked to stigmatization. Sante Publique 2016 Jun; 28: S127–S134. 28155782

[39] Mannan M , Mamun A , Doi S , Clavarino A . Is there a bi‐directional relationship between depression and obesity among adult men and women? Systematic review and bias‐adjusted meta‐analysis. Asian J Psychiatr 2016 Jun; 21: 51–66. 27208458 10.1016/j.ajp.2015.12.008

[40] Mannan M , Mamun A , Doi S , Clavarino A . Prospective associations between depression and obesity for adolescent males and females—a systematic review and meta‐analysis of longitudinal studies. PLoS ONE 2016 Jun; 11: e0157240. 27285386 10.1371/journal.pone.0157240 PMC4902254

[41] Keeter S , Miller C , Kohut A , Groves RM , Presser S . Consequences of reducing nonresponse in a national telephone survey. Public Opin Q 2000 Summer; 64: 125–148. 10984330 10.1086/317759

